# Femtosecond laser-assisted fabrication of chalcopyrite micro-concentrator photovoltaics

**DOI:** 10.3762/bjnano.9.281

**Published:** 2018-12-12

**Authors:** Franziska Ringleb, Stefan Andree, Berit Heidmann, Jörn Bonse, Katharina Eylers, Owen Ernst, Torsten Boeck, Martina Schmid, Jörg Krüger

**Affiliations:** 1 Leibniz-Institut für Kristallzüchtung, Max-Born-Str. 2, D-12489 Berlin, Germany; 2 Bundesanstalt für Materialforschung und -prüfung (BAM), Unter den Eichen 87, D-12205 Berlin, Germany; 3 Helmholtz-Zentrum Berlin für Materialien und Energie, Hahn-Meitner-Platz 1, D-14109 Berlin, Germany; 4 Fakultät für Physik und CENIDE, Universität Duisburg-Essen, Lotharstr. 1, D-47057 Duisburg, Germany

**Keywords:** chalcopyrite, femtosecond laser patterning, laser-induced forward transfer, micro-concentrator solar cell, photovoltaics

## Abstract

Micro-concentrator solar cells offer an attractive way to further enhance the efficiency of planar-cell technologies while saving absorber material. Here, two laser-based bottom-up processes for the fabrication of regular arrays of CuInSe_2_ and Cu(In,Ga)Se_2_ microabsorber islands are presented, namely one approach based on nucleation and one based on laser-induced forward transfer. Additionally, a procedure for processing these microabsorbers to functioning micro solar cells connected in parallel is demonstrated. The resulting cells show up to 2.9% efficiency and a significant efficiency enhancement under concentrated illumination.

## Review

### Introduction

In the field of renewable energies, the largest growth by far on a global scale in 2015/2016 took place in photovoltaics. However, the share of renewables in total energy consumption has recently increased only moderately, despite an enormous growth in the area of renewable energies. A major reason for this is the persistently strong increase in total energy demand [1]. This underlines the importance of the improvement of existing solar cell concepts and technologies in order to meet the high demand for low-cost solar power.

**Figure 1:**
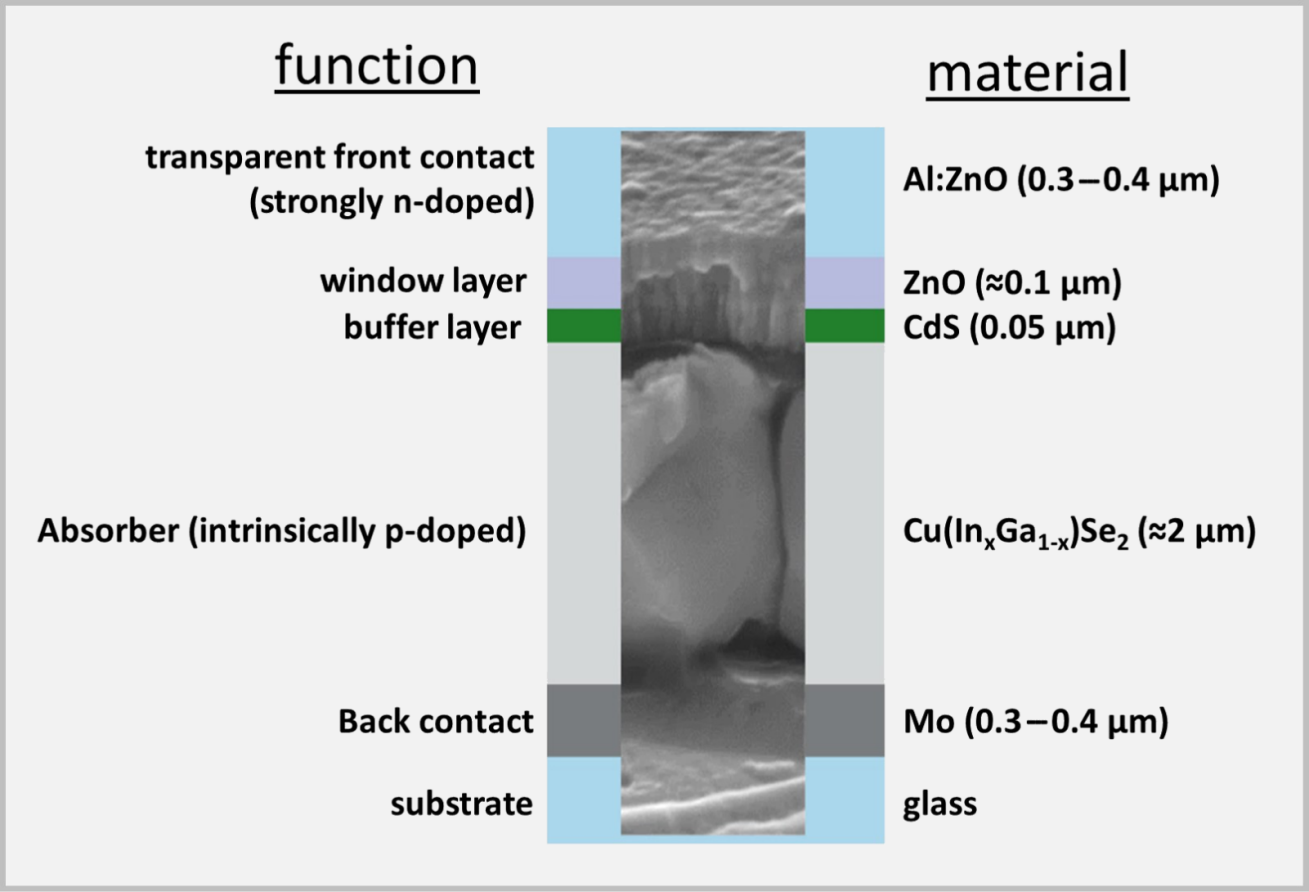
Design of a planar CIGSe solar cell.

In the present review, we provide an overview about research carried out on micro-concentrator solar cells – a new cell concept that has been emerging in recent years – using Cu(In,Ga)Se_2_ (CIGSe) as absorber material. The review focuses on two different laser-based fabrication methods for microabsorbers. In thin-film photovoltaics, Cu(In,Ga)Se_2_ (CIGSe) solar cells with an efficiency record of 22.9% for planar cells [2] and 19.2% for sub-modules [3] are among the leading technologies. Figure 1 shows the structure of a planar CIGSe solar cell representing the current state of the art.

**Figure 2:**
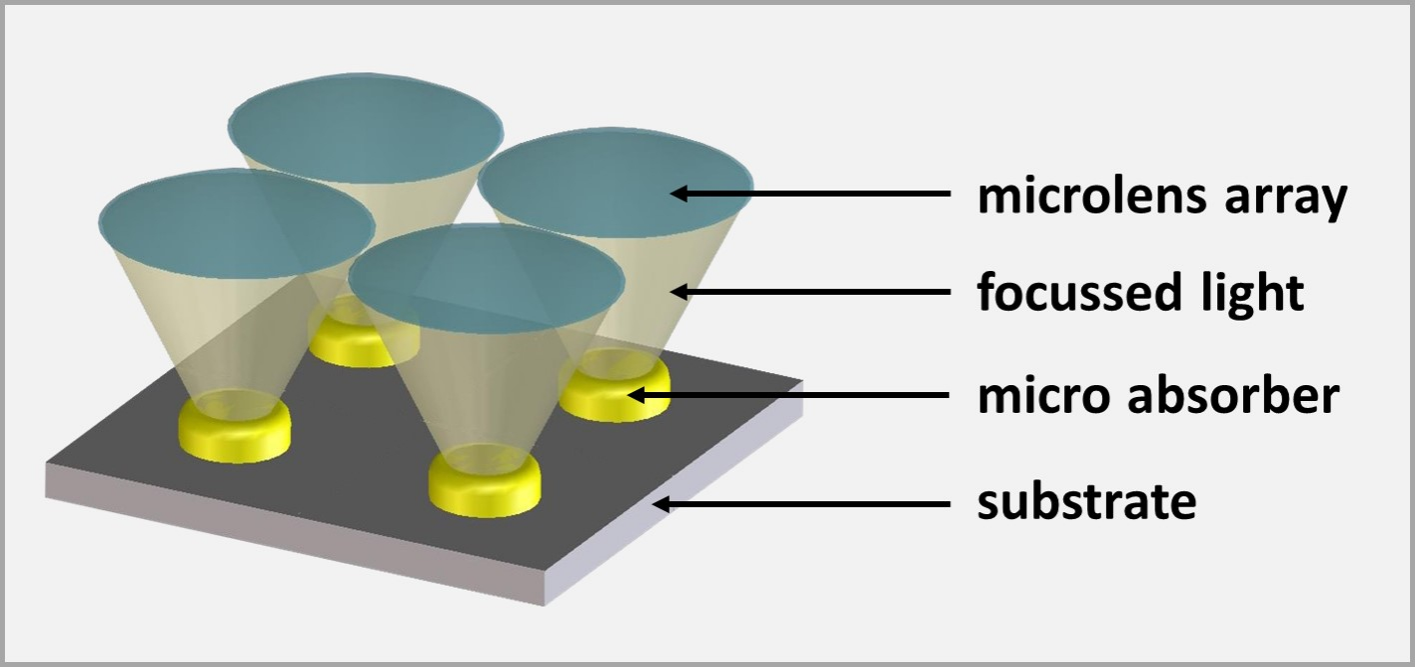
Scheme of the micro-concentrator solar cell concept.

The electric back contact (molybdenum) covered with the highly-efficient light-absorber (CIGSe) on top is deposited on a carrier material (glass). A buffer layer (CdS), a window layer consisting of an intrinsic ZnO layer (ZnO) and an aluminum-doped ZnO layer (Al:ZnO) as transparent front contact are located above the solar absorber. Since the CIGSe absorber is produced from highly demanded raw materials such as indium, which is also used for the production of light emitting diodes and flat screens, strong efforts are taken to improve cell efficiency and to develop material-saving fabrication processes and cell concepts. Among other things, current research aims to use light more efficiently through photonically active nanostructures, such that the layer thickness of the approximately 2 µm thick, planar absorber can be reduced (advanced light management) [4–5]. Another approach for saving raw material whilst enhancing the cell efficiency is the concept of CIGSe micro-concentrator solar cells. Instead of planar absorbers, the cells comprise only small absorber structures such as lines or dot-shaped islands, onto which the incident light is focused by microlenses. In Figure 2, this principle is illustrated for the case of dot-shaped solar cells.

Concentrator photovoltaics (CPV) require significantly less absorber material and, at the same time, the concentration of light allows for a more efficient energy conversion. The material saving potential for a squared array of microabsorbers can be estimated from the ratio of the area of one absorber island and the squared distance between the islands. For typical geometries, i.e., absorber island diameters between 40 and 100 µm and a distance of 500 µm, more than 97% of the material can be saved.

Since thickness and weight of concentrator cells both scale with the cell size, flat-plate-like weight and form factors can be realized by downsizing classical CPV to the microscale. Since the amount of heat, which is concentrated on each cell, is lower than for macroscopic concentrators, the system has a better heat dissipation, which has a positive effect on efficiency and lifetime [6–9]. In addition, the small dimensions allow for exploring unconventional architectures and for revisiting optical concepts that have been discarded in the past because of high material cost and optical absorption limits. Meanwhile, fully automated planar micro-tracking systems with less than 2 cm thickness have been developed, which may open up an avenue towards planar rooftop CPV [10]. Taken together, these aspects make micro-scale CPV an attractive approach for next-generation solar cells, which has been explored for several years [11]. These benefits of micro-CVP have to be traded off against the cost of additional components (e.g., lens arrays) and new production technologies for the assembly of microabsorber arrays.

Also for CIGSe, the concept of micro-concentrator solar cells has received increasing attention in recent years. On the one hand, studies were published in which CIGSe micro solar cells have been produced by top-down approaches such as etching or shading of flat absorbers. Paire et al. achieved an absolute increase in efficiency of 5% with 475 suns [12] and Reinhold et al. up to 4.8% for point-shaped cells [13]. They demonstrated that the increase in cell efficiency and the optimum light concentration varied with the size of the cells. Lotter et al. achieved a CIGSe micro cell efficiency as high as 22.5% under 77 suns by selective etching of the front contact layers [14]. While these studies show the efficiency potential of the micro-concentrator concept for CIGSe solar cells, the aspect of material saving was not considered in the chosen top-down approaches. Recently, bottom-up approaches were developed to locally deposit metallic precursors for CIGSe microabsorbers. By means of electrodeposition, the groups of Paire [15] and Sadewasser [16] successfully deposited linear and dot-shaped precursors and processed them to solar cells.

Here, we focus on reviewing two different femtosecond laser-based, material-saving approaches to produce CuInSe_2_ (CISe) and CIGSe microabsorbers. Several studies ranging from the fabrication of metallic precursors for absorber fabrication, their transformation to microabsorbers, processing to functioning solar cells up to their characterization both under standard conditions and concentrated illumination are summarized here comprehensively and illustrate the challenges and opportunities of the novel approaches to realize this cell concept.

The first approach for microabsorber fabrication summarized here is based on the growth of metallic precursors (indium islands) on laser-structured substrates (molybdenum on glass) by means of physical vapor deposition (nucleation approach). The second method presented is based on laser-induced forward transfer (LIFT). In this method, laser radiation is used to transfer parts of a donor film (copper, indium, gallium) from a transparent carrier medium (glass) to an acceptor substrate (molybdenum on glass) in a spatially controlled manner.

In the first part of this review regarding the fabrication of metallic precursors both approaches are discussed separately. The resulting challenges to process the precursors to microabsorbers and to produce functioning solar cells from these, however, were solved in an analogous manner and are thus summed up in a following joint part, which deals with the characterization of the resulting cells under different lighting conditions.

### Fabrication of metallic precursors

#### Nucleation approach

**Figure 3:**
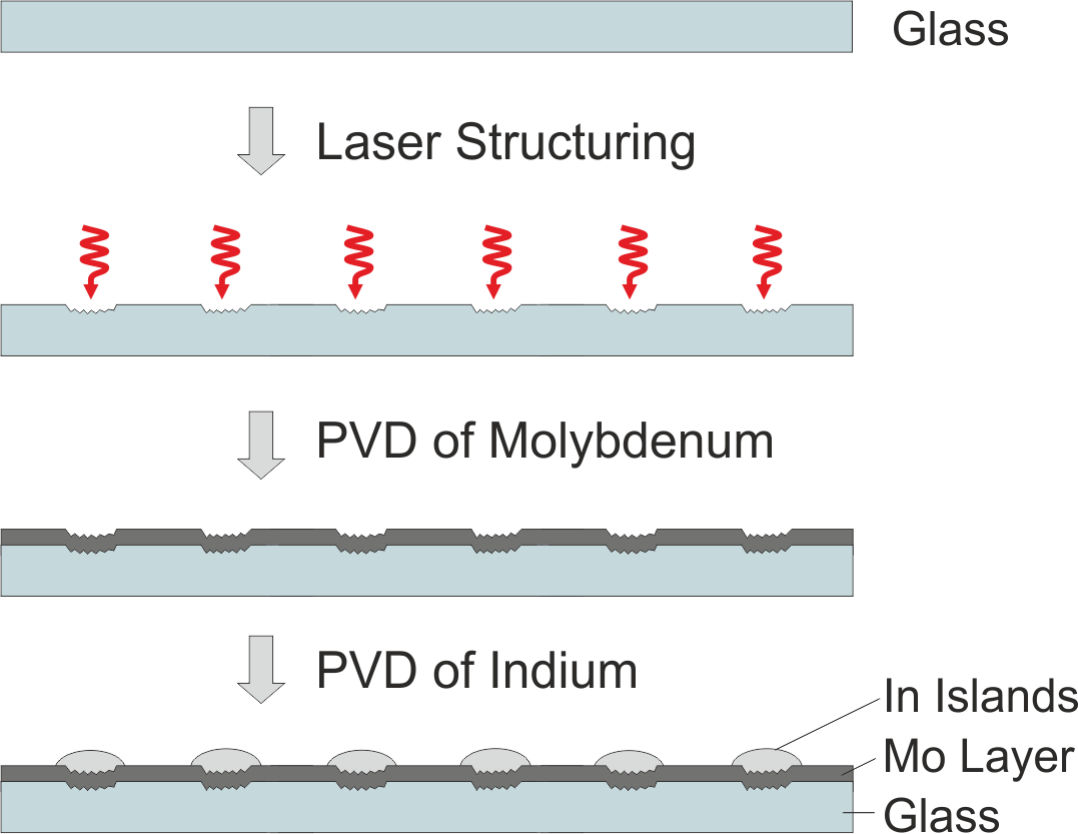
Schematic representation of ordered indium island growth on fs-laser structured, molybdenum-coated glass. Reprinted with permission from [17], copyright 2017 Elsevier.

The nucleation approach is based on the arrangement of metallic precursors by island growth on laser-structured substrates. Indium has a strong tendency towards island growth during physical vapor deposition (PVD) on molybdenum substrates. On smooth molybdenum surfaces, indium islands nucleate with random spatial distribution. Indium prefers to accumulate on rough areas. Hence, preferential island nucleation can be induced by local surface roughening. Existing indium islands then act as a material sink for further indium adatoms, such that they accumulate material and keep growing as long as further indium is deposited. At the same time, further island nucleation is suppressed in the vicinity of an existing island due to the constant depletion of freely diffusing indium. The radius around each island within which further nucleation is suppressed extends up to several hundred micrometers, depending on the experimental conditions [17]. The schematic process of indium island growth on molybdenum-covered glass substrates that were structured by a femtosecond (fs-)laser to induce nucleation at predefined locations is depicted in Figure 3. Here, the process is initiated by the laser structuring of the glass, followed by PVD of molybdenum and subsequently indium.

**Figure 4:**
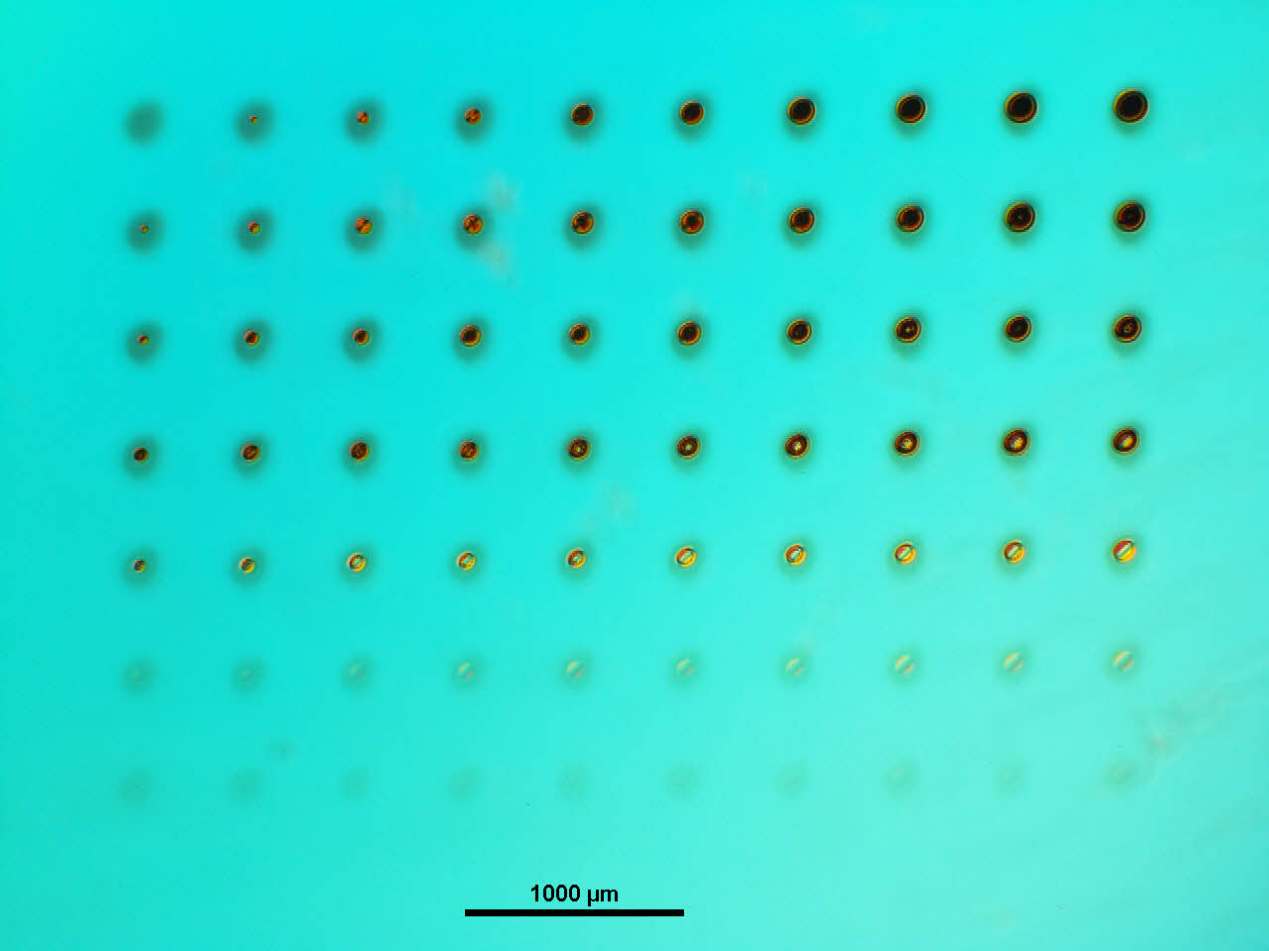
Optical micrographs of fs-laser-treated glass. For each line, the number of pulses per spot, *N*, is constant. From top to bottom, *N* amounts to 1000, 300, 100, 30, 10, 3 and 1. The peak laser fluence *F* varies from 1.24 J/cm^2^ (left) to 3.03 J/cm^2^ (right).

In other experiments, the glass substrates were PVD-coated with a molybdenum layer prior to the fs-laser treatment and the PVD of indium [18]. In both cases (fs-laser structuring of either glass substrate or molybdenum film), the resulting substrate surfaces were roughened or, upon harsher laser treatment, even exhibited crater-like depressions at well-defined spots. Figure 4 shows an optical micrograph (OM) of an array of laser-generated material modifications on glass, whereby pulse number and peak fluence of the laser (30-fs laser pulses at 800 nm center wavelength and 1 kHz repetition rate) were varied along rows and columns of the array.

**Figure 5:**
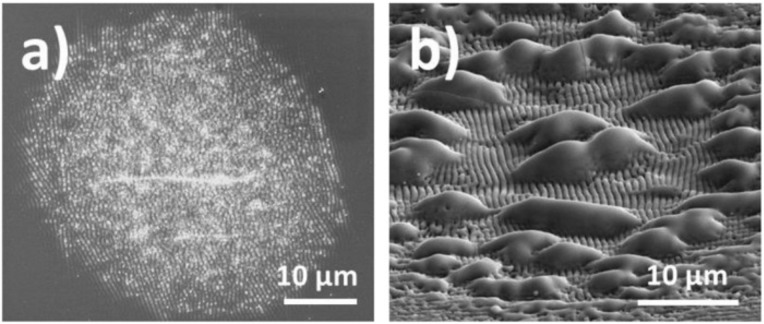
Scanning electron micrographs of laser-induced modifications on glass. Laser parameters: *F* = 1.63 J/cm^2^, *N* = 100 (a); 1.83 J/cm^2^, *N* = 30 (b). SEM tilting angle 0° (a), 52° (b).

The series of spots at the surface illustrates, that a stronger surface modification or even the formation of a crater can be achieved by increasing the number of laser pulses per spot as well as by increasing the laser fluence (energy density). Selected scanning electron microscopy (SEM) images of laser modifications on glass, which were recorded at tilting angles of 0 and 52° with respect to the surface normal, are depicted in Figure 5.

Figure 5a shows a laser spot with slight surface roughening that increases towards the center. Using a somewhat higher laser fluence, pronounced laser-induced periodic surface structures (LIPSS [19]) and round melting features form on the glass surface (Figure 5b). The LIPSS with periods in the sub-micrometer range are generated via intra-pulse scattering and interference of the fs-laser radiation at the roughened glass surface, leading to the spatially modulated deposition of energy in a shallow near-surface layer and, finally, to periodic material removal [20]. The micrometer-sized melting features supposedly arise from heterogeneities of the glass composition affecting the local optical and thermo-physical properties during the multi-pulse irradiation.

**Figure 6:**
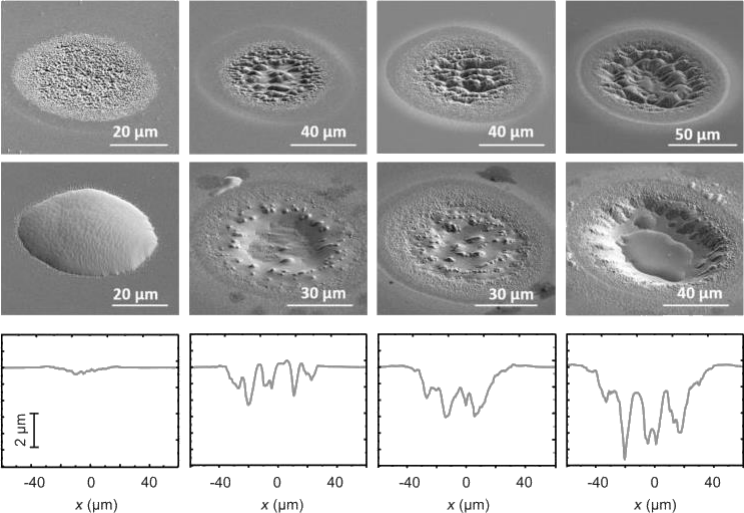
Scanning electron micrographs of individual laser-generated ablation spots on glass (top row) and corresponding profilometric cross sections (bottom row). Spots after deposition of molybdenum and indium (middle row). Laser parameters from left to right: *F* = 1.63 J/cm^2^, *N* = 100; *F* = 1.83 J/cm^2^, *N* = 30; *F* = 1.83 J/cm^2^, *N* = 100; *F* = 2.04 J/cm^2^, *N* = 100.

Figure 6 shows SEM images of individual laser spots on glass (top row) and their corresponding profilometric cross sections (bottom row). In the middle row, the spots are depicted after subsequent deposition of molybdenum and indium. The spots were created by applying different pulse numbers *N* and laser fluences *F* (from left to right: *F* = 1.63 J/cm^2^, *N* = 100; *F* = 1.83 J/cm^2^, *N* = 30; *F* = 1.83 J/cm^2^, *N* = 100; *F* = 2.04 J/cm^2^, *N* = 100).

For all depicted laser spots, the laser-generated surface structures constitute a diffusion trap for evaporated indium during the PVD process. The fact that the strongest indium accumulation occurs at the spot centers, which exhibit the highest roughness, indicates that the island growth is driven by the condensation of indium in the capillary-like structures. For the desired growth of flat and homogeneous indium islands, the data shows that a moderate roughening of the glass/molybdenum substrate surface, such as depicted in Figure 6, left column, provides the best results. Here, an indium island with a height of 2.6 µm and a diameter of 45 µm has grown on the glass/molybdenum substrate (Figure 6, left column, middle) on a laser-induced ablation spot in glass (Figure 6, left column, top) with a depth of about 300 nm in the center and a roughness *R*_a_ of about 25 nm averaged over the whole area (Figure 6, left column, bottom).

**Figure 7:**
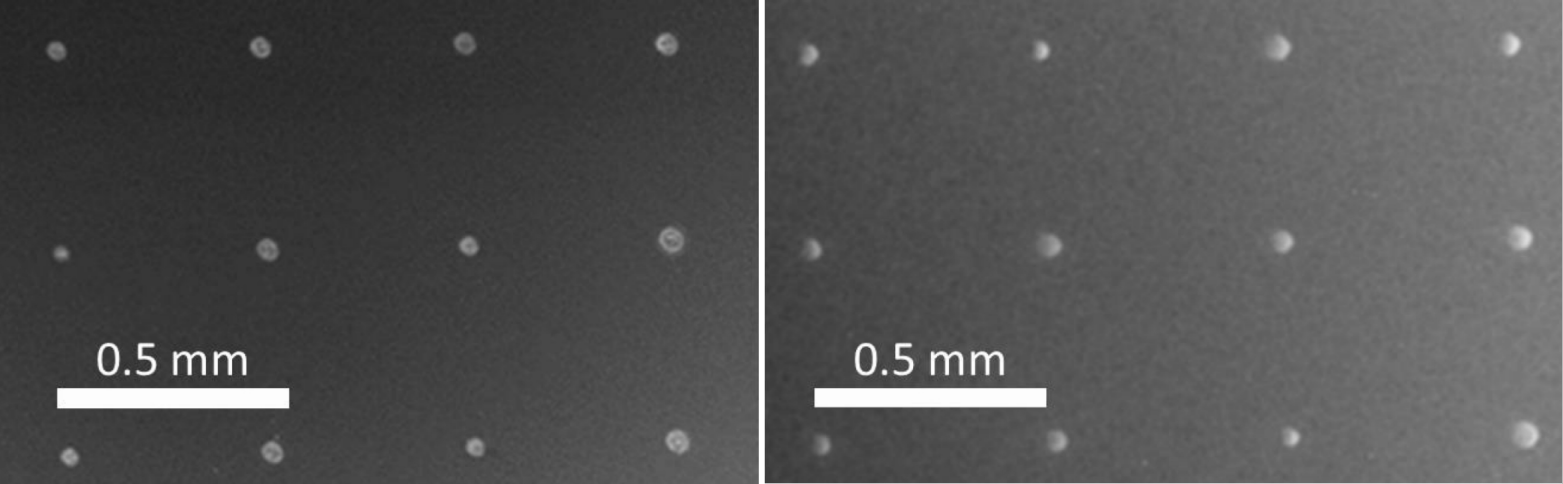
Optical micrographs of a laser-generated spot array on glass (left) and a corresponding array after PVD indium island growth (right).

In general, the diameter of indium islands, the geometrical aspect ratio and the nucleation density of indium islands all depend on the deposition rate and substrate temperature of the indium PVD process. In order to grow indium islands of well-defined size and aspect ratios and also for realizing arrays of specific spacings without undesired interstitial island formation, the island density and morphology had to be optimized through systematic examination of varying growth conditions. For the PVD process, the variation of temperature and deposition rate provided the insight that island distance and size increase with increasing substrate temperature. This can be intuitively understood by the higher mobility of the indium atoms diffusing on the substrate. At the same substrate temperature, a higher island density was observed by increasing the indium deposition rate. This is in line with the classical nucleation theory according to which the formation of stable nuclei depends on a critical (material specific) nucleus size. The shape of indium islands and the associated contact angle were significantly influenced by the temperature during PVD. At higher temperatures the islands became flatter, probably due to the decrease in surface tension of the liquid indium. The deposition rate of indium, however, had little influence on the contact angle of the islands. By optimizing the growth conditions, it was possible to determine parameters (ca. 500 °C substrate temperature and 0.3 Å/s deposition rate) at which suitable indium island (precursor) dimensions were achieved [17]. Figure 7 displays the result of the optimization process for an array of 500 µm spacing.

The optical micrograph on the left shows an array of laser spots on glass. The PVD of a 400 nm thick Mo back contact layer followed by indium island growth (at 500 °C substrate temperature and 0.3 Å/s indium deposition rate) led to an array of indium islands at the predefined positions (Figure 7, right). Obviously, no indium islands can be found at positions other than at the fs-laser irradiated spots, i.e., interstitial island formation was suppressed.

In contrast to indium, gallium showed a lower tendency for island growth and wetted the entire surface under all applied deposition conditions, such that a significant wetting layer formed in addition to gallium islands. Due to the different temperature dependence of surface mobility and adsorption–desorption equilibria, a sequential PVD process turned out necessary for the growth of (In,Ga) islands, whereby indium islands were grown first, onto which gallium was subsequently deposited. Optimum gallium deposition conditions were found to be a substrate temperature of ca. 400 °C and a deposition rate of 0.15 Å/s. Despite preferential aggregation of gallium at the existing indium islands, an additional gallium wetting layer was always observed. In order to avoid the undesired formation of a thin CuGaSe_2_ layer connecting the separate CIGSe islands after processing, this gallium wetting layer was removed by a mild reactive ion etching step in Ar^+^ plasma.

#### LIFT approach

**Figure 8:**
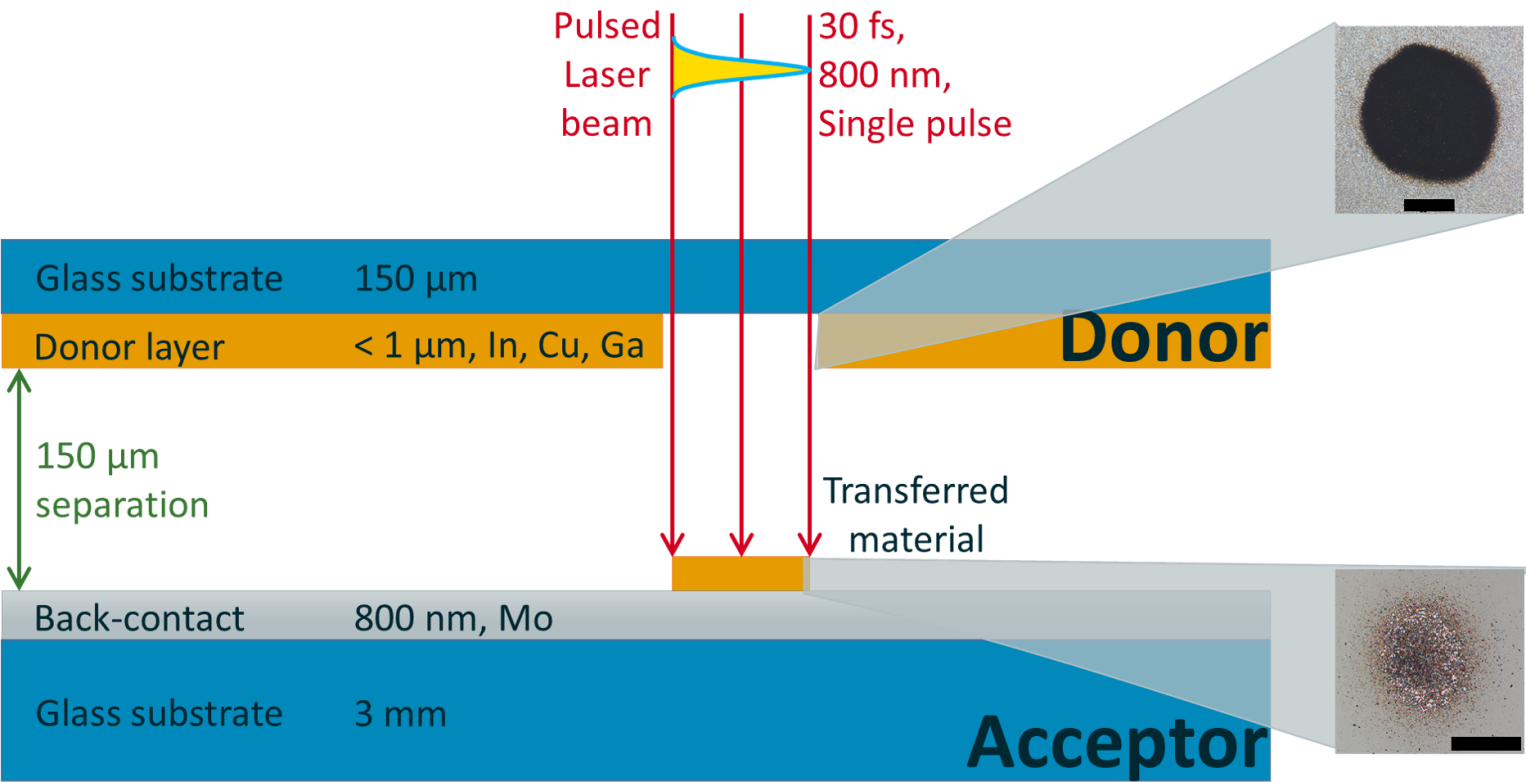
Scheme of laser-induced forward transfer. The scale bars in the OM insets on the right-hand side correspond to 50 µm. The OM pictures show the partial transfer of a 150 nm thick indium layer. Reprinted with permission from [22], copyright 2017 Springer Nature.

The second approach presented here for the production of precursor structures for CIGSe microabsorbers is the so-called laser-induced forward transfer (LIFT). In this method, a single laser pulse is used to transfer a part of a donor film located on a transparent substrate onto an acceptor substrate in a spatially structured manner. Prior to the laser treatment, the donor material is deposited on a donor substrate (glass) by means of PVD. The LIFT process was first introduced in 1986 for the transfer of copper onto a silicon substrate using excimer-laser radiation [21]. The experimental setup for the LIFT investigations is schematically shown in Figure 8.

The laser was operated at 30 fs pulse duration and 800 nm center wavelength. Single laser pulses were focused on the glass–metal interface to transfer material from the donor substrate onto the molybdenum back contact of the future solar cell. The distance between donor and acceptor was set to 150 µm. Single layers of copper (10–100 nm thickness) or indium (150–1000 nm thickness) as well as combined copper–indium layer stacks (210–1010 nm) were used as donor materials. Copper was first applied by PVD in all layer stacks because it has a significantly higher melting point than indium [22].

In a first set of experiments, LIFT of pure copper with varying layer thickness (10–100 nm) was investigated. The donor layers were irradiated by single pulses with fluences in the range of 0.8–7.8 J/cm^2^. The threshold for the LIFT decreases with decreasing copper layer thickness. In the case of the thinnest copper layers (10 nm, 20 nm), the laser energy is absorbed over the entire layer, resulting in a spray-like transfer of material. No transfer was achieved for a 100 nm thick copper layer. In the layer thickness range of 30–60 nm, a transfer was obtained, which was fragmented to varying degrees, depending on the laser fluence. In contrast to copper donor layers, indium can also be transferred from thicker donor layers. This is presumably due to different layer homogeneity (closed copper layers vs granular indium layers) and different thermo-physical properties of the materials. The quality of the transfer is generally comparable to that of copper [22]. On the right side of Figure 8, optical micrographs of LIFT results of an indium film are shown. While the upper image depicts the hole in the indium donor layer of 150 nm thickness after a single laser pulse irradiation at a peak laser fluence of *F* = 7.8 J/cm^2^, the lower image displays the spray-like deposit on the acceptor side.

**Figure 9:**
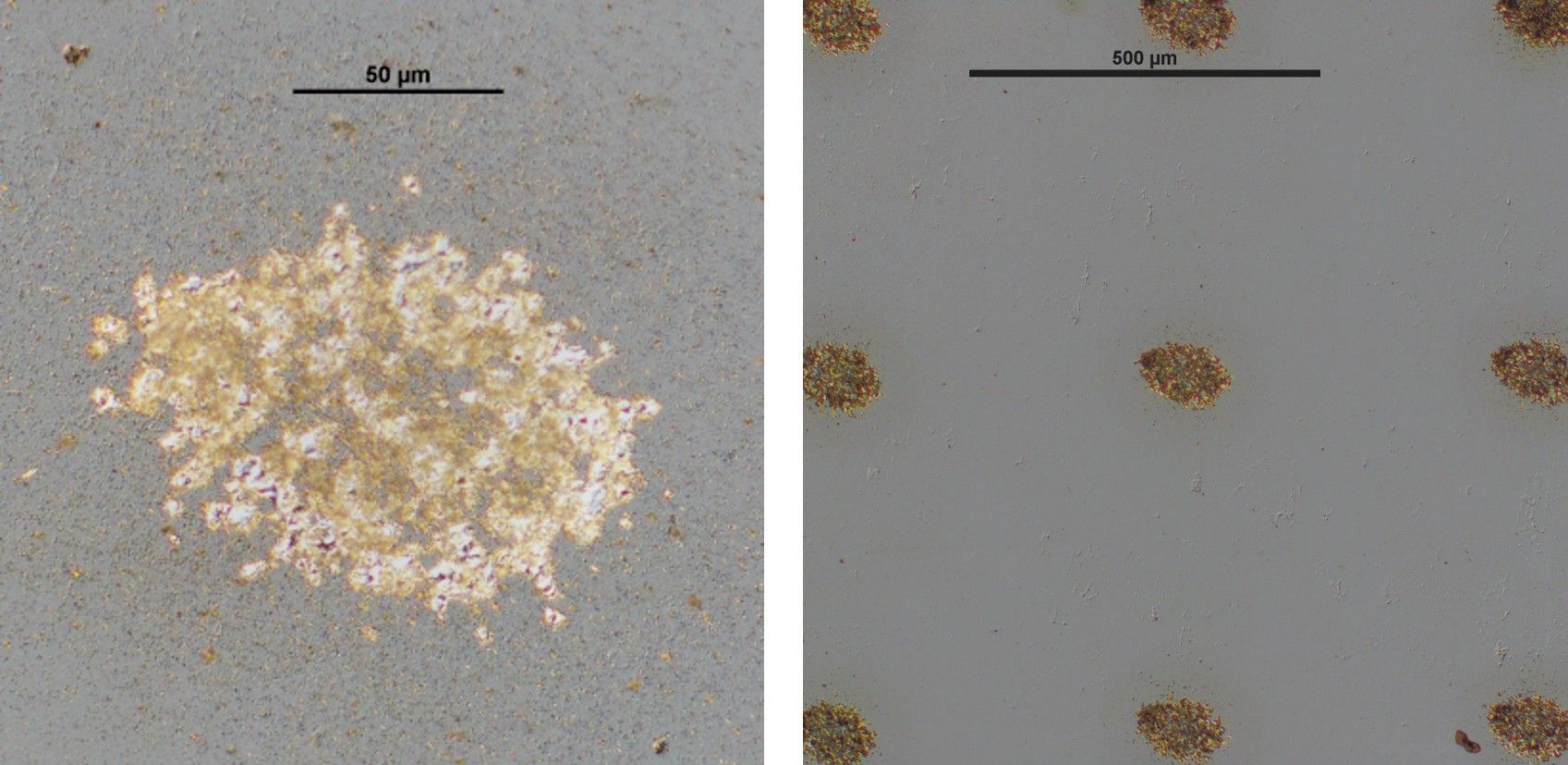
Optical micrographs of LIFT deposits on molybdenum on glass. Cu–In donor layer: 20 nm copper, 200 nm indium. *F* = 7.8 J/cm^2^. Left: single deposit with higher resolution. Right: array of deposits.

Figure 9 provides the result of a LIFT process of a combined copper–indium donor layer consisting of a 20 nm thick copper layer and a 200 nm thick indium layer. In contrast to pure copper or indium films [22], more homogeneous and compact deposits are formed on the acceptor using the combined copper–indium donor layer (Figure 9, left). Figure 9, right, shows the possibility of arranging compact copper–indium deposits in a freely selected array geometry by LIFT. Here, a square pattern of deposits with a distance of 500 µm was chosen, which is compatible with a potential geometry for micro-concentrator solar cells.

The LIFT deposits were characterized with respect to morphological and chemical homogeneity by using SEM and energy dispersive X-ray spectroscopy (EDX). It was investigated whether oxygen and carbon accumulations were formed within the transferred material, since these could have a negative effect on the resulting microabsorbers. Neither carbon enrichment nor indications for increased oxidation were detected. The thickness of the deposits was measured by optical microscopy with focus variation. The (average) thickness of a typical copper–indium LIFT deposit (Figure 9, right) is below 1 µm, which is in line with the targeted value for a whole CIGSe absorber of 1–2 µm (see Figure 1) [22].

#### Processing to microabsorbers

**Figure 10:**
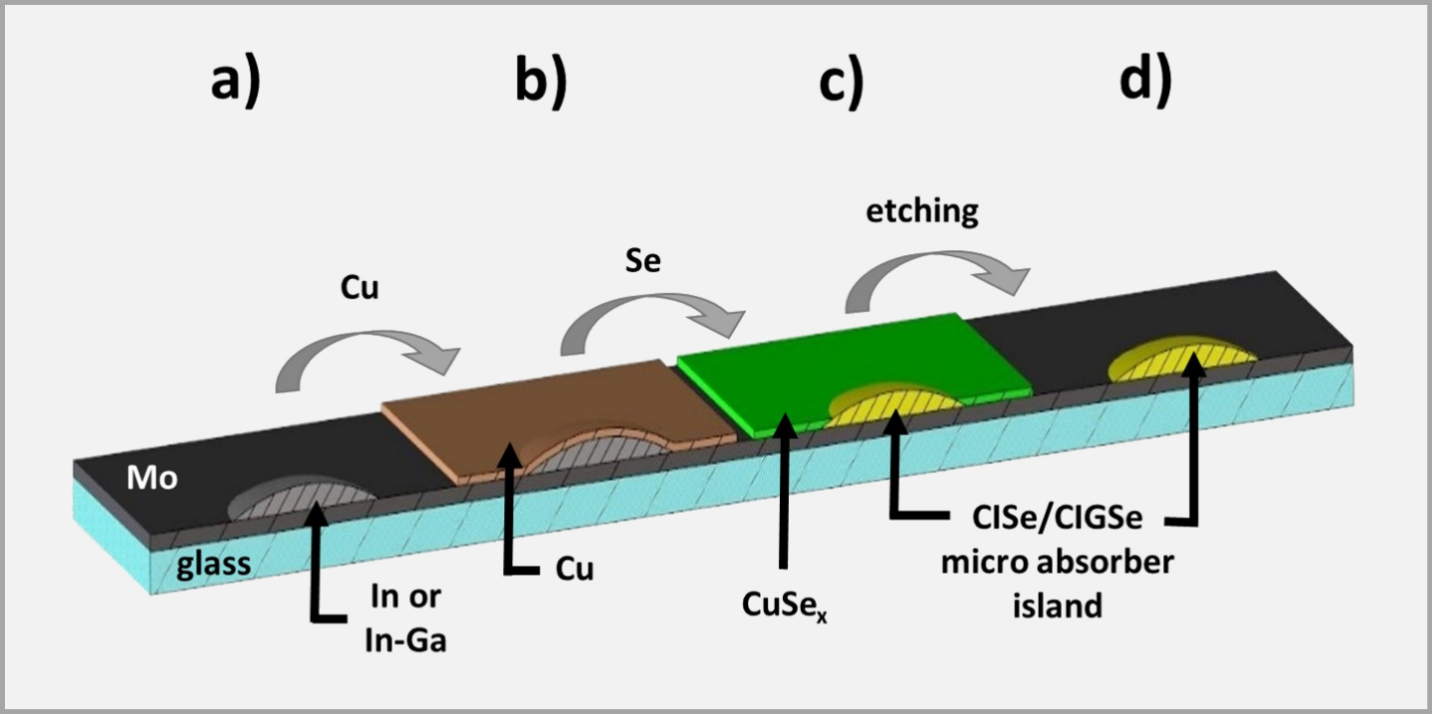
Scheme of the bottom-up process for the preparation of CISe or CIGSe microabsorbers via the nucleation approach. a) Bare In/In–Ga island on a molybdenum-coated substrate, b) In/In–Ga island coated with a flat copper layer, c) sample after selenization process, d) CISe/CIGSe absorber after etch removal of CuSe*_x_*.

**Figure 11:**
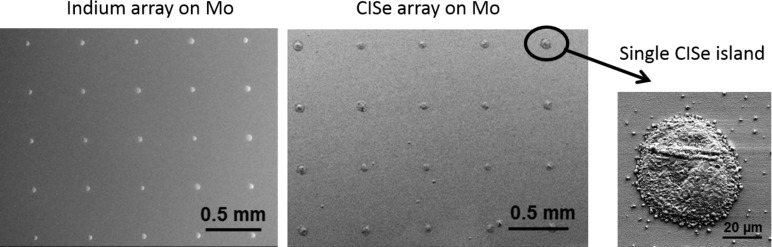
Processing of In precursor islands prepared by the nucleation approach (left) to CISe micro absorbers (middle and right).

In order to process In or In–Ga islands grown by the nucleation approach to CISe or CIGSe microabsorbers, the steps depicted in Figure 10 were applied. Since copper always formed flat layers regardless of the substrate temperatures investigated (from room temperature up to 500 °C), a copper layer of 500 nm thickness was routinely deposited onto In/In–Ga islands at room temperature (Figure 10b). As a consequence, covering copper selenides formed during the subsequent selenization step in-between and also partially on top of the absorber islands (Figure 10c). These compounds were removed by selective etching in 10% aqueous KCN solution for 3 min (Figure 10d). This etching step is also a standard procedure for the removal of copper selenides in conventional CIGSe production. Figure 11 shows an indium island array prepared by the nucleation approach before (left) and the corresponding CISe array after (right) the processing steps described above. More details for the absorber formation from In islands, in particular on the influence of the Cu layer thickness, can be found in [23].

In the LIFT approach, all metal precursors were transferred from the donor layer to the acceptor substrate in a single transfer step. Therefore, no additional metal deposition step was required. However, selenization and removal of potentially formed copper selenides by etching in KCN was carried out analogous to the processes for the precursors from the nucleation approach.

Selenization was realized by rapid thermal processing either in a graphite box at near ambient pressure for CISe samples from the nucleation approach and all samples from the LIFT approach, or in an ultrahigh-vacuum chamber with a directed selenium beam for CIGSe samples from the nucleation approach. In both cases, the temperature protocol comprised an annealing step at around 200–250 °C followed by a high-temperature plateau in the range of 500–560 °C (see [24] for details). It turned out that the homogeneity of the absorbers, in particular in the case of CIGSe samples, was sensitive to the selenization parameters. This effect was particularly significant for CIGSe samples from the nucleation approach the homogeneity of which was clearly enhanced when the high-temperature plateau was increased from 500 to 560 °C.

#### Processing to solar cells

**Figure 12:**
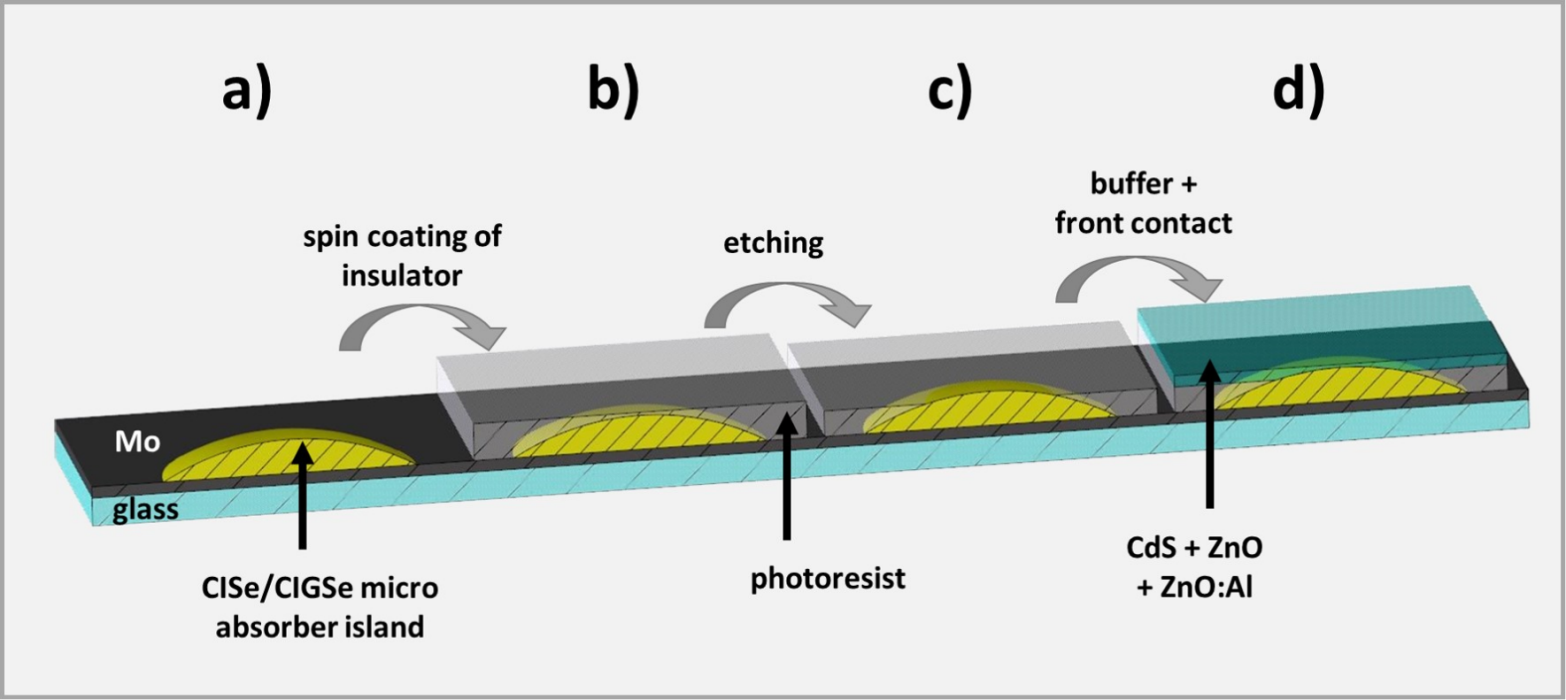
Scheme of the process for manufacturing solar cells from microabsorbers. a) CISe absorber, b) spin coating of photoresist (insulator), c) reactive ion etching in Ar^+^ plasma, and d) addition of CdS and ZnO buffer layers and Al:ZnO front contact.

From microabsorbers that are regularly arranged on a common substrate, a monolithic system of microcells, which are connected in parallel, can be fabricated. A process to realize such a system is illustrated in Figure 12.

**Figure 13:**
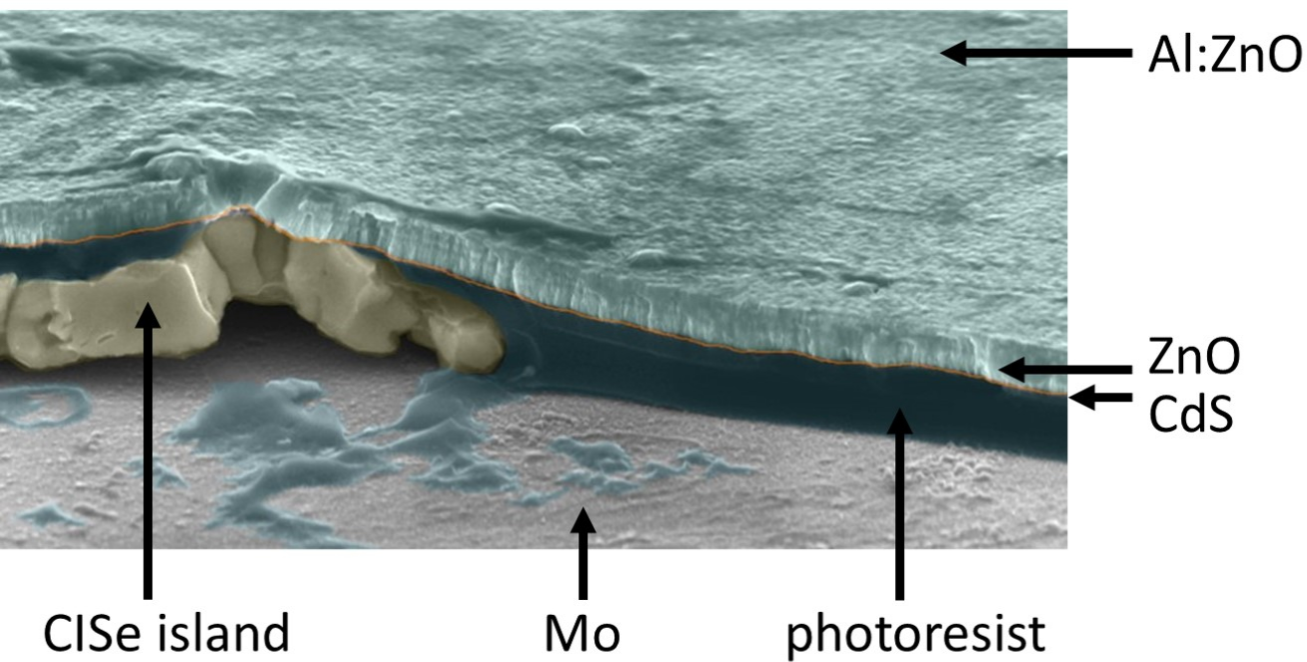
Cross section of a CISe micro absorber island after processing to a micro cell imaged by tilted-view SEM. Note that the different materials were artificially post-colorized to enhance their visibility. The height of the CISe absorber is ca.1 µm.

Before buffer layers (CdS, ZnO) and front contact (Al:ZnO) can be deposited, the electric insulation between back and front contact in-between the microabsorbers must be ensured. Due to its high (thermal) stability, ease of use and low electrical and high thermal conductivity, the photoresist SU8 was used for this purpose. In order to apply the photoresist, a precursor solution was distributed evenly on the sample via spin coating (Figure 12b). Subsequently, this solution was photochemically converted into SU8 and cured by means of thermal treatment. This procedure comprised a pre-bake (3 min at 95 °C), an UHV treatment (exposure for 10 min to UHV light of 385 nm wavelength), a post-bake (1 min at 65 °C followed by 2 min at 95 °C) and finally a hard bake (3 min at 200 °C). To guarantee electric connection between the front contact and the CIGSe islands, it is necessary to remove the uppermost part of the SU8 layer, such that the top of the islands is exposed. Upon choosing an appropriate initial viscosity, the SU8 layer is significantly thicker on the substrate than on top of the islands. Therefore, a mild treatment by reactive ion etching (22 min at 250 W in Ar atmosphere), for example, is sufficient to uncover the islands while keeping the molybdenum substrate isolated (Figure 12c). Finally, the buffer layers (CdS, ZnO) and the front contact (Al:ZnO) were deposited (Figure 12d). CdS was applied by a wet-chemical bath deposition. Subsequently, ZnO and finally Al:ZnO layers were created in a sputtering process. Details for CdS and ZnO/Al:ZnO deposition can be found in [25]. Figure 13 shows an SEM image of the edge of a CISe micro island, which has been processed according to this procedure, i.e., in a state corresponding to Figure 12d.

The advantage of the spin-coating approach is that the insulating layer not only covers the molybdenum substrate, but also fills cavities or holes that might form sporadically within the islands and that would lead to power leakage in a lithography-based isolation approach.

### Characterization of solar cells

The solar cells were characterized under AM (air mass) 1.5 standard test conditions and at elevated light concentration factors up to 100 suns. For the latter purpose, a concentrator sun simulator was used that also fulfilled AAA conditions (highest spatial uniformity, temporal stability and spectral match with the AM 1.5 sun spectrum). In order to achieve measurably high currents and to facilitate electric wiring, approximately 25 to 100 micro solar cells were simultaneously measured in a parallel interconnection scheme. For efficiency calculation, the active absorber areas were estimated by either calculation from single microabsorber sizes or by optical microscope measurements. The absorber areas estimated for the different absorber fabrication approaches were (0.00125 ± 0.00007) cm^2^ for CISe islands from the nucleation approach, (0.00145 ± 0.00008) cm^2^ for CIGSe islands from the nucleation approach and (0.0019 ± 0.00003) cm^2^ for CIGSe islands from the LIFT approach. Errors in area measurement are given and directly translate into uncertainties of the final current density and cell-efficiency values. Further errors may arise from the fact that the active absorber area may still be smaller than the measured one. For all three types of locally grown micro solar cells, working devices were obtained. Table 1 summarizes the solar cell parameters determined under 1 sun illumination. The *IV* measurements under 1 sun illumination were depicted in [24].

**Table 1:** Solar-cell parameters at 1 sun illumination compared for micro cells fabricated from the different local absorbers and the corresponding planar reference cells.

	*j*_SC_ (mA/cm^2^)	*V*_OC_ (mV)	FF (%)	η (%)

nucleation CISe	27.5 ± 2.3	295	36	2.9 ± 0.2
nucleation CIGSe	29.7 ± 2.3	132	36	1.4 ± 0.2
LIFT CIGSe	2.9 ± 0.2	145	36	0.15 ± 0.02
planar reference nucleation CISe	33 ± 5	406 ± 50	39 ± 5	5.9 ± 0.5
planar reference nucleation CIGSe	36 ± 4	505 ± 20	45 ± 2	8.5 ± 0.4
planar reference LIFT CIGSe	34 ± 2	425 ± 10	48 ± 4	8.1 ± 0.6

Astonishingly, the open-circuit voltage (*V*_OC_) for the CISe microcells is more than twice as high as the one reached by the CIGSe absorbers. According to the dependence of band-gap energy on the Ga content, the opposite behavior would be expected. This observation points to the fact that the intermixing of In and Ga in the quaternary compounds has still to be improved. In contrast, the short-circuit current per active area (*j*_SC_) is almost comparable for CISe and CIGSe micro solar cells from the nucleation approach, but a factor of ten lower for the CIGSe microabsorbers fabricated via LIFT. The lower current densities achieved for the absorbers from LIFT fabrication can be attributed to a remaining lack of compactness of the absorbers leading to lower carrier generation and extraction. The fill factor (FF) is comparable for all three types of absorbers. Overall, an efficiency (η) of 2.9% for CISe islands from the nucleation approach, of 1.4% for CIGSe islands from the nucleation approach and of 0.15% for CIGSe islands from the LIFT approach was demonstrated. Planar reference cells were fabricated in a sequential process as well, and the precursor stacks were designed according to the bottom-up growth process. This means the same element sequence was chosen for direct comparison, which however, does not correspond to an optimization for planar absorbers. The corresponding efficiencies of the planar references were 5.9% for CISe by nucleation, 8.5% for CIGSe by nucleation and 8.1% for CIGSe by LIFT. The efficiencies given were obtained as an average of measurements on 16 individual solar cells with 0.5 cm^2^ size each. For the CISe microcells obtained from the nucleation approach the efficiency amounts to 50% of the planar reference under 1 sun illumination. Given the facts of efficiency enhancement under light concentration and of more than 97% material saving, a relative increase in efficiency per volume by more than 46% can be expected.

**Figure 14:**
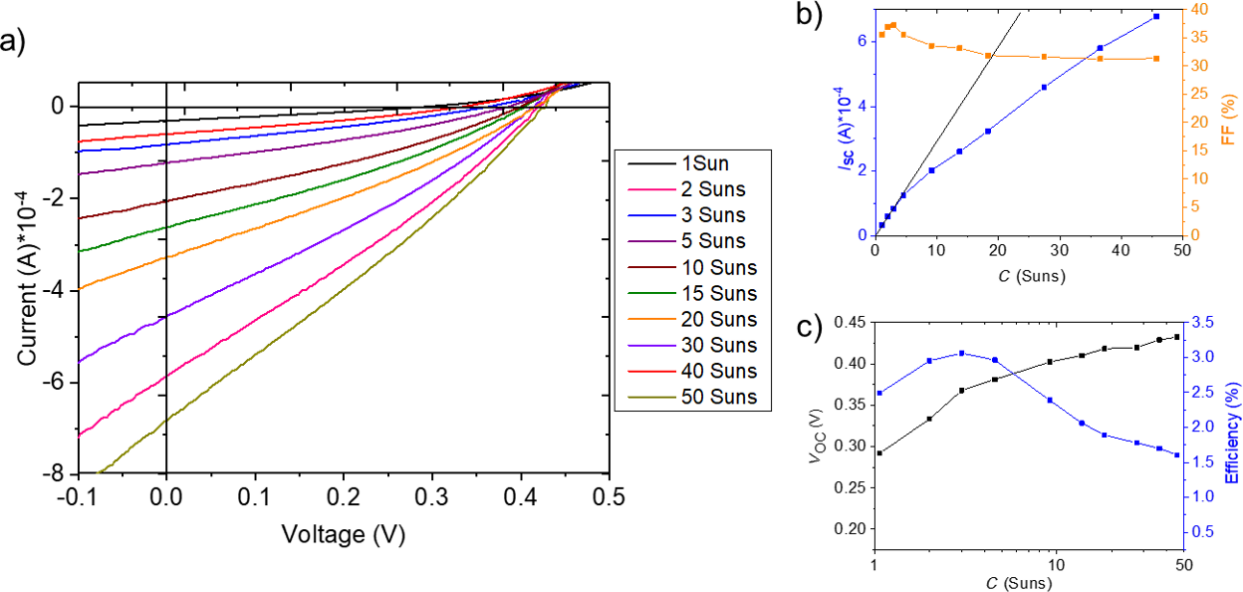
Electrical characterization with different light concentration factors for a CISe microcell from the nucleation approach: a) *IV* curves, b) *I*_SC_ and FF, c) *V_oc_* and η.

**Figure 15:**
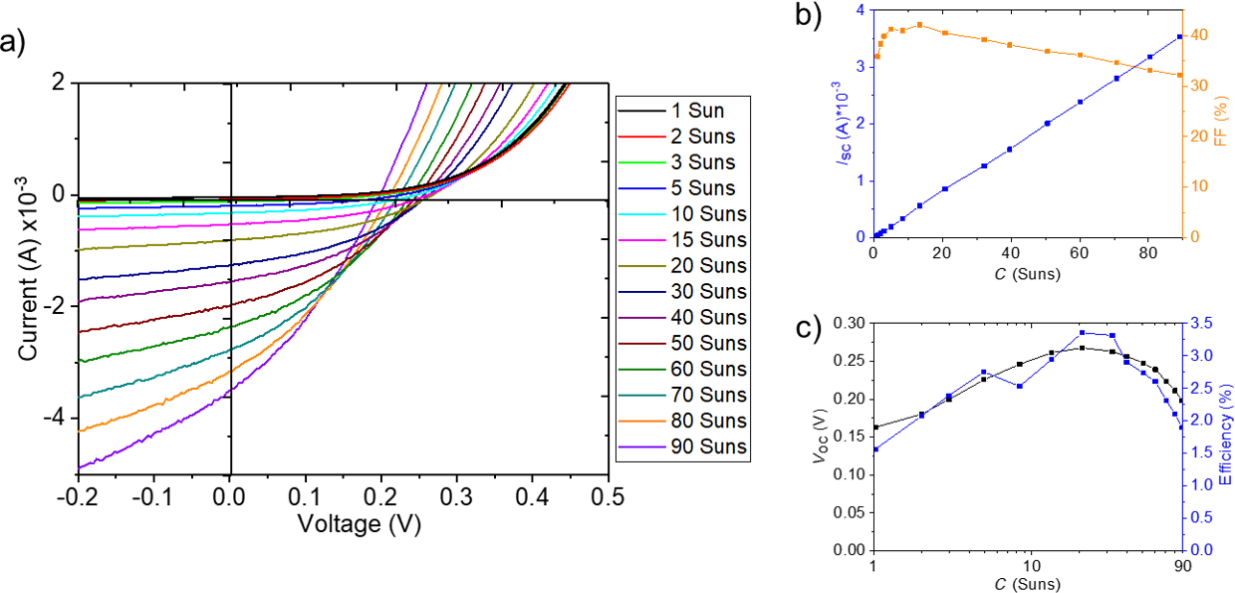
Electrical characterization under various light concentration factors for CIGSe micro cell from nucleation approach: a) *IV* curves, b) *I*_SC_ and FF, c) *V*_OC_ and η.

**Figure 16:**
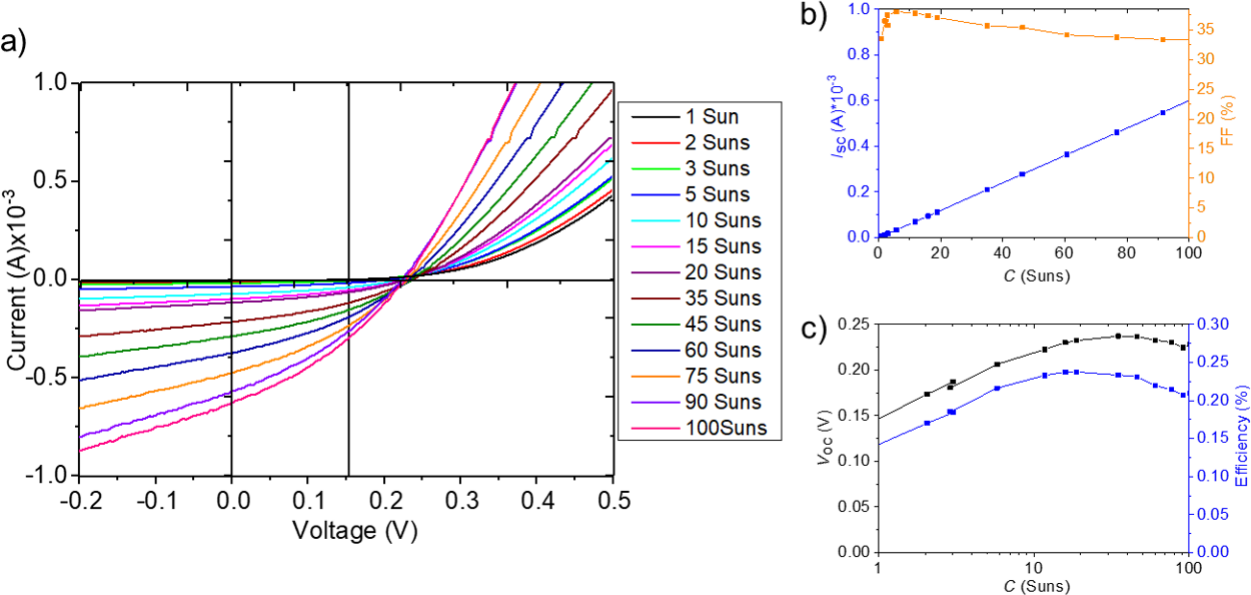
Electrical characterization under various light concentration factors for CIGSe micro cell from LIFT approach: a) *IV* curves, b) *I*_SC_ and FF, c) *V*_OC_ and η.

The results of measurements under enhanced illumination intensities are shown below in Figures 14–16 for CISe and CIGSe islands from the nucleation approach, and CIGSe islands from the LIFT approach, respectively. In an ideal concentrator solar cell, the current increases linearly with the concentration factor. This is, however, due to the increase in incident power upon concentration. Thus, both factors cancel each other when it comes to efficiency calculation. The net efficiency enhancement results from the fact that, in addition, the open-circuit voltages rises logartihmically with the concentration factor, which can be deduced from the diode equation:

[1] $$I=I_{\text{L}}-I_{0}\left[\exp\left(\left(qV\right)/\left(nk_{\text{B}}T\right)\right)-1\right],$$

with *I* representing the total current, *I*_L_ the photo current, *I*_0_ the dark current, *q* the elementary charge, *k*_B_ the Boltzmann constant, *n* the diode quality factor and *T* the temperature. By solving for *V*_OC_ = *V*(*I* = 0) and performing the substition of *I*_SC_ = *I*_L_ with *I*_SC_·*C*, where *C* is the concentration factor and *I*_SC_ the short-circuit current we obtain:

[2] $$\begin{array}{c} V_{\text{OC}}\left(C\right)=\left(nk_{\text{B}}T\right)/q\cdot \ln\left(I_{\text{SC}}\cdot C/I_{0}+1\right)\\ \approx V_{\text{OC}}\left(C=1\right)+\left(nk_{\text{B}}T\right)/q\cdot \ln\left(C\right). \end{array}$$

This is inserted into the expression for the efficiency:

[3] $$\eta =\left(j_{\text{SC}}\cdot V_{\text{OC}}\cdot \text{FF}\right)/P_{\text{in}}.$$

With the incident power density *P*_in_ = *C*·1000 W/m^2^ and the enhancement in *V*_OC_ by (*nk*_B_*T*)/*q*·ln(*C*), the efficiency under concentration for an ideal cell translates to:

[4] $$\eta \left(C\right)=\eta \left(C=1\right)+\left(nk_{\text{B}}T\right)/q\cdot \ln\left(C\right)\cdot \left(j_{\text{SC}}\cdot \text{FF}\right)/P_{\text{in}}.$$

For the three cases of differently grown microabsorbers, we tested these expectations by investigating *IV* measurements with different values of the light concentration (*C*).

For CISe microabsorbers from the nucleation approach, Figure 14a depicts the development of *IV* characteristics from 1 to 50 suns. As Figure 14b illustrates in more detail, *I*_SC_ only experiences the predicted steep linear increase up to a concentration factor of three, which quickly decreases thereafter. Also FF is governed by a small peak around 3 suns before declining. *V*_OC_ follows a more steady increase of, in this case, logarithmic rise also at higher concentration levels, yet again with a peak at 3 suns, see Figure 14c. In consequence, the efficiency reaches its maximum at 3 suns already. The deviation from linear rise in current can be understood when looking at the development of series and shunt resistance as a function of the concentration. Both are decreasing with increasing illumination intensity, yet this happens faster for the shunt resistance as it can be deduced from the *IV* curves. A resulting effect is the drop of *I*_SC_ at higher concentration values.

Moving on to CIGSe microcells fabricated by the nucleation approach, we can, in contrast, observe from Figure 15b a perfectly linear increase in *I*_SC_ with concentration up to 100 suns. Yet, *V*_OC_ experiences a drop above 30 suns, as it can be seen from *V*_OC_(*C*) plot in Figure 15c. In combination with an even earlier decline of FF (Figure 15b), this behavior leads to an efficiency maximum at 20 suns.

An overall very similar behavior is found for CIGSe microcells fabricated by the LIFT approach, as illustrated by the results of *IV* measurements shown in Figure 16 and in [25]. Here, *I*_SC_ also increases linearly, but *V*_OC_ decreases above 30–40 suns, leading, together with a quick decrease in FF, to a maximum in efficiency at 20 suns.

For both cases of CIGSe microabsorbers, shunt and series resistances drop with similar slopes in a double-log plot, which, however, is more detrimental for the higher shunt resistance. This is consistent with a drop in FF but a linear increase in *I*_SC_.

A remaining question is why *V*_OC_ starts to deviate from the expected logarithmic increase at a certain concentration level. One possible explanation is the experimental approach chosen here: The entire micro solar cell array is illuminated with enhanced light intensity, which leads to heating of the whole assembly including the non-active areas, in particular at higher light concentration levels. In a microconcentrator device, however, light will be focused on the absorber area only and the design will benefit from improved heat dissipation. A further enhancement can thus be expected in the final device. In the configuration investigated here, the highest efficiencies were 3.06% at 3 suns for CISe microcells from the nucleation approach, corresponding to a relative enhancement of 6% compared to illumination at 1 sun. An efficiency of 3.36% at 20 suns was achieved for CIGSe microcells from the nucleation approach, i.e., a relative enhancement of 138%. CIGSe microcells from the LIFT approach reached 0.237% at 20 suns and thus a relative enhancement of 60% compared to illumination at 1 sun. These enhancement factors constitute a promising starting point for future research from which efficiency maxima at elevated concentration factors and higher efficiencies can be expected.

### Conclusion

The promising new solar cell concept of micro CPV was addressed in this review using CISe and CIGSe microabsorbers. A particular challenge for a material-efficient fabrication of such microcells is the local bottom-up growth of absorbers. For this purpose, two laser-based methods were applied, namely the nucleation approach and the LIFT approach. In both cases, metallic precursors were created, site-controlled via femtosecond-laser treatment, which were subsequently processed to microabsorbers. For further processing to microcells, a pathway was demonstrated, in which an isolation concept based on spin coating was applied. The advantage of this approach is that imperfections can be compensated, since the spin-coated photoresist insulates any potentially occurring irregularities such as microcavities. The microcells were connected in a parallel manner and exhibited efficiencies between 0.15% and 2.9% under 1 sun illumination. Under concentrated illumination, significant efficiency enhancements could be achieved. These results constitute a promising step towards the maturation of this cell concept.
